# Developing Future Physical Education Teachers' Intercultural Competence: The Potential of Intertwinement of Transformative, Embodied, and Critical Approaches

**DOI:** 10.3389/fspor.2021.765513

**Published:** 2021-12-02

**Authors:** Mariana Elisabet Siljamäki, Eeva Helena Anttila

**Affiliations:** ^1^Faculty of Sport and Health Sciences, University of Jyvaskyla, Jyväskylä, Finland; ^2^Theatre Academy, University of the Arts Helsinki, Helsinki, Finland

**Keywords:** equality, diversity, intercultural competence, physical education, teacher education, Finland, transformative learning, embodied language learning

## Abstract

**Purpose:** This article is based on a study that explored learning processes related to intercultural competence of PE teacher trainees. The context of the study was the Faculty of Sport and Health Sciences at the University of Jyväskylä, Finland. The study was conducted in connection to two courses that focused on equality in physical education and sport in 2020–2021.

**Methods:** Adopting an interpretive, as well as a critical approach, the authors focused on how the students described their conceptions and learning experiences. Based on their analysis they have then aimed to shed light on how interculturality, equality, equity, and diversity may be addressed in higher education in a more profound manner. The students' accounts were analyzed first through an open reading and subsequently through a more critical lens. The analysis was supported by theories of transformative learning, embodied learning, and intercultural education.

**Results:** Students' initial interest toward equity, equality, and interculturality seemed to expand during the courses. They increasingly reflected on the complexity of these issues and discussed the widening professional responsibilities of future PE teachers in promoting equality and supporting pupils in cultural heterogeneous classes. Discussions and practical activities that involved emotional and embodied elements seemed to be important in facilitating their learning processes. However, it is difficult to foresee how permanent the changes in their habits of mind and subsequent actions are.

**Discussion:** The authors suggest that embodied, practical approaches where the student is fully engaged in the learning process, and where conceptual, reflective, emotional, and affective levels are connected, may be a key in developing teachers' intercultural competence. They also suggest that it is crucial to revise higher education curricula from the perspectives of intercultural competence and structural inequality. In addition to separate courses, equality, equity, and diversity should be seen as red threads throughout higher education.

## Introduction

It is largely acknowledged that future teachers will work in educational institutions that are increasingly diverse, both culturally, linguistically and in relation to, for example gender. Our study is situated in Finland, which, amongst many other countries has become more heterogeneous for example, through increased immigration. At the end of 2020, a total of 444,031 people with a foreign background lived permanently in Finland, which is 8% of the population. Every tenth child under school-age has foreign background, as many as every fourth in Greater Helsinki (Statistics Finland, 2020).^1^ Increasing diversity is also reflected in the National Core Curriculum for Basic Education (2014), as it emphasizes changing societal conditions. According to the goals of the curriculum different identities, languages and religions live in parallel and interact with each other.

However, in practice there is much to do before these aims can be achieved for all pupils. When early school leavers with immigrant backgrounds are compared to Finnish-born youth, they have more difficulties in transitioning to upper secondary education (Kalalahti et al., 2017). In addition, immigrant-origin youngsters have reported a wide range of experiences of discrimination (e.g., Zacheus et al., 2017; European Union Agency for Fundamental Rights, 2018). Moreover, a report on “Being Black in EU” (2018) reveals that among the 12 countries in the survey, Finland topped the list regarding perceived racial discrimination as well as harassment speech and gestures. Appearance, such as the different kinds of dress, is also one of the reasons for harassment (Mannila, 2021). These results indicate that there is still much more work ahead in working toward equality and social justice for all.

The national curriculum (2014) defines the goals of physical education (PE), which is an important part of Finnish schools and a compulsory subject in basic education. PE is a school subject that aims to contribute to pupils' holistic well-being by supporting their physical, social and psychological functional capacity and positive body image. In this article our aim is to shed light on the learning processes that are related to intercultural competence in PE, and thus support future PE teachers' professional competence in the increasingly complex educational contexts.

Intercultural competence, thus, can be considered as an area of growing significance as societies are becoming more diverse. Diversity is a multifaceted phenomenon that entails gender, class, and (dis)abilities in addition to cultural aspects such as ethnicity or religion. However, it seems fair to say that all societies that value social justice and human rights need to address the diversification of cultural values and invest in intercultural competence in all public services, including education. In our view, education is one of the most crucial contexts where true difference in this regard can be made. We have approached this topic in our earlier research and considered teachers as “frontline agents of integration” (Anttila et al., 2018). Another way to put this is to consider all teachers as language teachers and to see language as a broader phenomenon than verbal, spoken language (Siljamäki and Anttila, 2020). Moreover, physical education is most often a holistic activity that involves embodied collaboration and non-verbal communication (Thorburn and Stolz, 2017). Thus, in the context of physical education (PE) young people can meet, get to know, and learn to respect each other. For this to happen, PE teachers need to be well-prepared to teach culturally diverse groups and expand their professional roles and tasks. However, little is known about the ways that physical educators see their professional roles and goals in culturally heterogeneous classes or how they perceive pupils with different cultural backgrounds (Barker, 2019). Physical educators need to be aware of their privileged positions (Nieto and Bode, 2008, p. 4–5; Barker, 2019) and grasp the complexity of interculturality in education and in larger society. In this study, the complex concept of interculturality is dynamic and relies on a hybrid and contextual notion of culture. Interculturality is understood as the relations that exist between culturally diverse individuals or groups,^2^ and it refers to the relations that exist within society between diverse majority and minority, and also to ethnicity, language, religious denomination, or nationality (Cushner, 2011; Dietz, 2018). The concept of cultural diversity is not unproblematic and has been criticized for placing too much emphasis on seeing differences as categorical and permanent without taking into account the intertwining of differences and the dynamism of cultural and social aspects (e.g., Nieto, 2010). In our research, we seek to address the above criticism and understand that individuals who share the values of the same culture also have individual beliefs and behaviors connected to many other areas of life (see Nieto, 2010, p.18–22).

This article is based on a study that examines how the development of PE students' intercultural competence can be supported. We will focus on this topic through analyzing PE teacher trainees' preconceptions on teaching culturally heterogeneous groups, and investigating how their views might have changed during two courses that aimed at developing intercultural competence. In this article we will present initial findings and interpretations on data collected between fall 2020 and spring 2021.

## Key Concepts and Theoretical Framework

There are many concepts associated with intercultural education, such as intercultural communicative competence (Byram, 1997), cultural self-awareness and intercultural sensitivity (Bennett, 2009), all of which refer to the ability to face cultural diversity and differences in a positive way (Jokikokko, 2010). The main concept for this study, intercultural competence, refers to awareness of one's own, culturally related thought patterns, attitudes, language-aware teaching, the ability to work with people from different cultures on a situation-by-situation basis and interaction skills (Byram, 1997; Friedman and Berthoin Antal, 2005; Jokikokko, 2010; Nastasi, 2017). Intercultural situations pose challenges to commonly shared principles and practices of working together (e.g., Salo-Lee, 2007; Jokikokko, 2010, p.30, 59). Interaction skills include, in addition to spoken language, the ability to negotiate issues and to act in conflict situations, as well as non-verbal and embodied dialogue (Byram, 1997; Friedman and Berthoin Antal, 2005). Jokikokko (2010, p.72) summarizes that even though intercultural competence is related to specific skills and knowledge, it can be seen more as a holistic approach to issues, and as an ethical orientation to people (see also the concept of competence used by Spitzberg and Cupach, 1984).

In PE, intercultural competence is concretely related to how the teacher supports equal opportunities for pupils to participate in embodied practices, regardless of their cultural background, language, and religion. Moreover, in the context of PE, the notion of embodiment is relevant, and needs to be considered carefully (Thorburn and Stolz, 2017). Teachers need to be aware about issues that may be culturally sensitive, including bodily interaction, touch, gender, cultural and religious interpretations of the body, as well as dress in physical education classes. However, within research on intercultural competence, embodiment has rarely been discussed in depth. We have touched the relationship between culture and embodiment, that is, the various views on the body and embodied activity, and related views like touching and clothing in our earlier research (Siljamäki and Anttila, 2020). It is clear that norms regarding the body and movement in culturally diverse settings cannot be dealt with in a preconceived way. This means that it is not possible to define individual views on these issues based on their cultural or ethnic background only. Each individual forms their personal conceptions throughout their life experiences, thus, teachers need to be sensitive to individual needs and views, and not to label students based on their ethnicity, for example.

The theory on transformative learning (Mezirow, 2009; Mezirow and Taylor, 2009; Mälkki, 2010) has a major role in the theoretical framework of our study. According to (Mezirow, 2009, 92) transformative learning refers to a process by which meaning perspectives and habits of mind that shape and delimit one's perception, cognition and feelings become more inclusive, discriminating, open, reflective and emotionally flexible. Habits of mind, once set, predispose our intentions, beliefs, expectations and purposes, and guide our choices and actions. These habitual ways of thinking, feeling and acting become articulated in a specific point of view that shapes a particular interpretation. The process of transformative learning begins when an individual encounters a disorienting dilemma that becomes a trigger for reflection. Reflection, thus, may emerge in connection to events or situations that bring our worldview and beliefs under question. This kind of transformation of a habit-of-mind, which allows people to navigate the world in a way that is “more inclusive, discriminating, open, reflective and emotionally able to change” may be sudden or cumulative (Mezirow, 2009, 92). From the individual's point of view, learning is a matter of creating meaning, which is by nature an interpretation of reality. In transformative learning, experience is interpreted from a new, consciously and more comprehensive perspective. Transformative learning can be distinguished from simply the acquisition of content knowledge or skills competence (Mezirow, 1997; Mezirow and Taylor, 2009). Transformative learning has also been discussed earlier in the studies in the context of PE teacher training. This approach can challenge the beliefs and the ways of seeing the nature of PE (e.g., Luguetti and Oliver, 2021).

Mälkki (2010) emphasizes the role of emotions and embodiment in transformative learning and supplements Mezirow's theory with neuroscientist Damasio's (1999, 2010) views on emotions and consciousness. She points out that reflection has been the buzzword of adult and higher education for the past decades, and that various practices for facilitating reflection have been developed and studied during recent years. However, educational contexts rarely involve disorienting dilemmas or conditions that trigger such profound reflection that may lead to transformative learning. Mälkki introduces the concepts of comfort zone and edge-emotions for depicting the emotional orientation of our thinking. The notion of edge-emotions refers to a situation where the continuity of meaning perspectives becomes challenged, and as a consequence, gives rise to unpleasant emotions that require the individual to leave the so-called comfort zone. Comfort zone refers to the pleasant experience of being able to make meaning unproblematically within the meaning perspective whereas edge-emotions refer to unpleasant feelings that emerge when our meaning perspective becomes questioned (Mälkki, 2010, p.30).

Embodiment, indeed, adds another layer to reflection and in learning even in higher education contexts. In her doctoral study, Sööt focused on the initial dance teacher preparation, trying to find a more profession-specific approach and procedures that support reflection in novice dance teachers. Sööt's study connected reflection and the somatic, embodied point of view to create a guided core reflection procedure (Sööt, 2018, see also Sööt and Anttila, 2018). The significance of embodiment in making ethical judgements in the context of music and dance teachers' education and expanding professionalism is a topic of another recent study (Sutela et al., 2021). We will return to the significance of embodiment in transformative learning and the notion of expanding professionalism in the results section.

## Context

The context of the study is the Faculty of Sport and Health Sciences at the University of Jyväskylä, Finland. The Faculty is the only University-level unit that offers PE teacher education in Finland. Although Finland can be considered one of the model countries for equality, a young person from an academic background is significantly more likely to participate in University education than a young person raised in a non-academic family (Nori, 2011; Käyhkö, 2015). The backgrounds of the students are not insignificant, because the University students are not individuals without social origin. The people in a privileged position often do not perceive or understand that different values related e.g., to cultures or religions may seem very foreign from one's own background. In the middle-class culture of society, the values can be defined as the “right” choices (Käyhkö, 2015). Since 2014, the programme has taken up the challenge of increasing diversity by offering a course that focuses on inter- and multicultural education, sexual and gender diversity as well as individual educational needs. In the autumn of 2020, the faculty introduced a new curriculum, in which the above-mentioned areas were strengthened with another course.

This study is conducted within these two courses, entitled Basics of equality and equity in physical education and sport (University of Jyväskylä Study Guide, 2020–2023a) and Equality and equity in teaching in physical education (University of Jyväskylä Study Guide, 2020–2023b), specifically on sections that focus on inter- and multicultural education. The first course took place in fall 2020. It was a 3-day intensive workshop, consisting of, for example, lectures, physical activities outdoors and indoors, as well as group discussions. The lectures dealt with concepts related to interculturality such as different definitions of culture, intercultural competence, racism, ethnocentrism and otherness as well as studies concerning the experience of harassment, discrimination and racism. The concept of embodiment and its significance in physical education was also reviewed. In addition, the course included various small group assignments where conceptual and practical contents were combined and where personal experiences, for example, regarding feelings of exclusion were discussed. Students also prepared poster presentations in small groups.

The second course took place in spring 2021 with lectures, activities on embodied language learning, approaches to adapted physical education, and so forth. The lectures concerned the learning of the Finnish language in multilingual classes, as well as issues to be taken into account in PE of Muslim pupils. Embodied language learning as a part of the course is related to language awareness at school. The National Core Curriculum for Basic Education of Finland (2014) emphasizes that every adult is a linguistic model, and a teacher of the language of the subject (s)he teaches. Embodied (or kinesthetic) language learning is a holistic process where movement, bodily sensations, and experiences are involved in learning (Anttila et al., 2018). A lecture on the equal consideration of Muslim pupils in PE was added to the course because some fears and skepticism toward Islamic religion and its values emerged in the students' preconceptions. The purpose of the lecture was to provide students the information about the religion of Islam and discuss concrete situations that should be considered in PE classes, when Muslim pupils are involved.

The courses are mandatory for all PE teacher trainees. The learning outcomes of these courses are designed to develop the ability to promote equity, inclusion and equality in PE; to increase awareness of the effect of own values, attitudes, cultural background and previous experiences in interaction; to increase awareness of emotions related to encountering diversity; to develop the ability to critically reflect own behavior related to equality, equity and human rights in the context of PE; and to deepen understanding of the meaning of embodiment in PE.

## Methodology

### Interpretive Research

Our methodological approach draws largely from interpretive research tradition that has been widely applied in, for example, arts education research since the late twentieth century (Wasser and Bresler, 1996; Eisner, 1998; Green and Stinson, 1999). Interpretive research focuses on meanings, and acknowledges researchers' positionality and situatedness. As interpretive researchers, we are not searching for a single truth, or even many truths. Instead, our interpretations are social constructions and tightly connected with our own experiences and (pre)conceptions of the phenomenon that we are researching. Our intention has been to read the data–in this case, students' accounts collected through online surveys and interviews–as openly as possible. However, we are aware that neither the data nor our readings are simple representations of a certain social reality that can be fully grasped through our research. Our interpretations rise from multiple, ever-changing realities, including our own. These realities are situated in societal and cultural conditions that are governed by ideologies, norms, and power structures.

We acknowledge the critique that has been posed toward qualitative research methodologies and social constructionism, including approaches to data collection and analysis (St. Pierre, 2011; Lather and St. Pierre, 2013; Lather, 2014; Denzin, 2015). Societal conditions are at play in many ways in what becomes said and told, and in how experiences become communicated through words. The process of negotiation of what students' words might mean takes place between us as researchers and authors of this article. Our choices on what words we choose when describing our interpretations, and which citations from students' accounts represent some part of social reality are influenced by what we already know, think, believe, and understand. We acknowledge that “how a thing gets inside the text is shaped by a politics of representation” (Denzin, 2015, p.200). We are also aware about and interested in the complex connections between experience and language, in how language shapes experience, and in how “meanings are always in motion, incomplete, partial and contradictory” (Denzin, 2015, p.200).

Our research interest is a complex combination of the need to understand, and the need to promote social justice, that is, emancipatory critical research interest (see Habermas, 1972). We are interested in both contesting our interpretations and our own preconceptions that, for example, leads us to think that the development of intercultural competence is connected to transformative learning. Thus, we move across paradigms, much like Star Brown describes: “Essentially, qualitative inquiry enabled me to explore meaning, and post qualitative analysis facilitated a study of how meaning was positioned within power structures.” (Brown et al., 2021, p.232). We also ask, are students telling us what we want to hear? How much of their accounts is socially constructed discourse that arises from their privileged positions and limited experiences in inter- or multicultural contexts? To what extent students' desire to meet other cultures can be seen from a personal enrichment perspective, and how can such a view be combined with a social justice perspective that may make it necessary to leave one's comfort zone?

### Methods, Procedures, and Research Questions

Having stated all of the above, our data collection process has been quite conventional and it has yielded student accounts in the form of verbal language. All participants of the study attended both above-mentioned courses. Mariana, one author of this article has developed and co-taught these courses with two colleagues of the faculty of sport and health sciences. Her teaching focused on interculturality in PE while her colleagues taught the other sections. In addition, all three teachers strived to ensure that the different sections formed a holistic understanding of equality.

In fall 2020, before the course started, all students (75) completed a preassignment and they returned their reflections to the learning platform. In this essay, they were asked to write about their previous experiences, preconceptions, and emotions regarding, for example, cultural diversity. Most students (64) gave permission to use their essays in our research. Each student wrote an approximately page-long reflection. In January 2021, before the beginning of the second course, all students (81) responded to a set of open questions, presented to them *via* an online survey tool. The questions concerned how students perceived their future work in teaching multilingual, culturally and religiously diverse groups. After the second course, they (80 students) responded to another set of open questions. Again, most students (72) gave consent to use their responses in our research. In addition, 10 students volunteered to participate in an interview in their own time. The interviews took place after the latter course and due to the Covid-19 pandemic, Mariana conducted interviews remotely. The interviews lasted an average of 50 min and yielded 206 pages of transcribed text. The aim of the interviews was to gain a deeper understanding of students' conceptions, learning experiences and further education needs.

The specific research questions are as follows: (1) How do the students describe their preconceptions regarding interculturality, language awareness, and equality? (2) How do the students reflect on their learning experiences during these courses? (3) How do students describe the change of their conceptions in the context of the courses? We have formulated these how-questions purposefully as we acknowledge that the way participants express their views may be as important as the actual content of the responses. Consequently, we have not conducted a conventional thematic or content analysis. Instead, we have created a descriptive report of student accounts, based on an open (vs. theory-driven) reading of the data. We are supporting this report with selected quotes. We present the report in three sections, where each section is connected to one of the research questions. The sections are followed by an interpretive synthesis where we connect the descriptive report with earlier research. In addition, we contest our reading of the students' accounts and raise some critical viewpoints, thus acknowledging that both our data and our analysis may be biased.

### Ethics

We recognize that the dual role of the teacher-researcher affects the research as a whole, and requires us to be as transparent as possible about research ethics and procedures. According to the ethical protocols of the University of Jyväskylä and the Finnish National Board on Research Integrity (2021), the students were provided with the option not to participate in the study. The participants were told that their identities would not be revealed at any stage of the study, including this article, and at all stages of the material acquisition their answers would be treated confidentially. Additionally, it was important to point out that their responses would not affect the course grade in any way, and that at any point, they had the right to withdraw their participation in the study without stating the reason for doing so, and without any consequences.

Being aware of the dual role of the teacher-researcher also in the analysis and interpretation of the material is important. Eeva, another author of this article did not know the participants and she read the material from an outsider's perspective. The dual role of a teacher-researcher may also have its advantages because almost all students in the course agreed to take part in the study, which can indicate trust in the teacher-researcher.

To protect anonymity, we do not provide information on an individual student's background. All the students who participated in the study were of Finnish origin and between the ages 22–34. In addition to studies in PE, 20 participants were studying another degree and many of them had already work experience in an educational field. The other participants were studying their first degree. Most of them studied for a second year in the Faculty of Sport and Health Sciences.

When analyzing the data we are not disclosing nor making interpretations based on students' gender. The issue of gender is complex and would be a relevant topic for research in itself. We have included a variety of citations from 39 students for strengthening the trustworthiness of our study.

## Results

### Initial Views, Attitudes, and Concerns: How Students' Background Shapes Their Expectations

This section focuses on the first research question and is based on students' preassignment essays and first survey. In general, most students^3^ seemed to be aware of the importance of equity, equality, and diversity before the courses. In their essays and the first survey they wrote about their own background from many perspectives. They described their family background, experiences of cultural encounters, or, on the other hand, how some had almost no experience of encounters with people from different cultures. Not surprisingly, own background was seen as having an important impact on the perceptions of different cultures, languages, religions, and equality. They described how their own bilingualism, working with culturally diverse groups, living in Helsinki, a city with cultural diversity, or for example, a spouse with an immigrant background have broadened their understanding of cultures and languages. Traveling abroad was also mentioned by many students as an important learning experience that helps to get to know cultures, as in this reflection:

*In my opinion, I am very open-minded about cultural diversity and I could consider the relationship with this even as one of my strengths as a future PE teacher. I have traveled really a lot around the world and have been interested in the cultures of different countries. Sure, there are so many different cultures in the world that you can't know or feel them but I think it helps so much to understand different people and how they act*.

In addition, some students had worked abroad and it had been an eye-opening experience for them. While working abroad the students learned to understand for the first time how much the norms, attitudes and religion of the community and the surrounding culture affect the individuals.

Some students pointed out that they come from a small, homogeneous locality, and because of that, they had not had any encounters with people from different cultures. This had not necessarily led to a negative attitude toward diversity, but the students wrote that it made an impact in at least some ways. One student wrote that, “*the attitude is a bit twofold due to my own background, I come from a small place where there have been no non-Finns.”* Another reported that, “*I moved from a small town to Helsinki three years ago and I can say that this was a significant moment in terms of accepting and especially appreciating diversity.”*

When describing their relationship to different cultures many students used words like openness, positivity and curiosity. Different cultures were considered interesting and enriching by most students. Statements like “*cultural diversity is a very fascinating theme”* and “*diversity gives a new perspective and an opportunity for a wider learning experience”* reflect this kind of attitude toward other cultures.

Many students also highlighted that cultural and linguistic diversity is an integral part of today's society and is in that sense normal, especially in metropolitan areas. Teaching culturally diverse classes was seen as a good opportunity for everyone to learn about another culture and language as well. In addition, the diversification of sports culture in physical education classes was generally considered as a positive phenomenon. Students as future PE teachers seem to find it interesting that pupils from different cultures could share their own ways of moving and teach new, different forms of sports for others. In the words of two students:

*In a culturally diverse class, there is the opportunity to become acquainted with different forms of exercise that are not necessarily practiced in all cultures*.

*I find it positive that we are able to go through even more diverse forms of exercise when we take into account the sports favored by different pupils. Many certainly have a sport form from their culture that others have not tried before*.

Many students described how, in their opinion, movement and physical education connect people from different backgrounds because in physical activity, there is no need to understand verbal language. For example, one student reflects that, “*I think that PE is one of the subjects that is easy to come to from different cultural and linguistic backgrounds. PE also gives a sense of community.”*

Somewhat more critical ideas toward intercultural encounters were also presented, although such accounts were exceptions. Few students wondered whether Finnish culture should always be flexible or whether interculturality could mean that other cultures could also be flexible as one student pondered:

*I am from an old-fashioned school; first comes to mind “in the country the way of the country,” but it is not so straightforward, different cultures have to be respected, but our culture should not be the only flexible party*.

Difficulties in understanding other cultures or religions also emerged in some preconceptions, several of them were related to the position of women in religion as in these reflections:

*I have a very open mind to different cultures and religions, but I must also say that sometimes it is difficult to understand something, no matter how you try. Perhaps these feelings and thoughts are precisely because we are accustomed to different habits here, and we often feel that those habits are the right ones. In particular, some radical differences in some matters may be difficult to understand (e.g., the status of women)*.

*I feel open-minded, but I still often find myself unconsciously prejudiced. Without generalizing any culture, there are features (in Islam, for example), where I feel the social status of women is completely incomprehensible to myself. However, I try to remember that that culture is divided into so many subcategories that I cannot say that the whole religion is certain*.

It is noteworthy that the same students seem to have a wide range of attitudes and thoughts, both open-minded and more skeptical views about different cultures or religions. This reflects how complex the phenomenon is. Negative attitudes of the students were due to, among other things, previously experienced situations at work or on how the media has treated people with an immigrant background or their religion. However, the most prominent attitude toward people from culturally and religiously diverse backgrounds seemed to be interest and positivity. Therefore, the students expressed their desire to act “in the right way” with culturally diverse groups. Many of them described fears of not being able to say the right words or correct terms, for example, related to cultures or religions or saying something offensive. This concern was common and came up in many preconceptions, of which the following quote is one example:

*From the point of view of future employment and why not even living, I am worried how I can cope with a little knowledge of culture or religion, because I do not want to offend anyone about it*.

Experiences of uncertainty were also accompanied by limitations brought by cultures and religions that are not known, e.g., what kinds of clothes students from different religions are allowed to wear, especially as swimwear. Participation in swimming and dance lessons, as well as dressing room conduct, were mentioned. To increase certainty, many hoped that they could learn about different cultures and religions or ideas and tips for “immigrants” PE. In the words of one student: “*It would be a good idea to review the different religions and cultures, as well as their main features and the tips on how to face and involve them.”*

Several students also expressed that they had experienced difficulties in verbal communication with culturally diverse groups, and in general, they seemed to be worried about their interaction skills, and highlighted the importance of language skills, as one student writes: “*Multiculturality is a challenging and scary thing for me due to linguistic reasons. I'm really bad at speaking English and I find that it limits my activities in all situations.”* The need to have more language courses in physical education teacher training in order to be able to communicate with their multilingual pupils was desired by some students. The wish for language teaching was related to the fact that the success of communication was considered central in the teaching of multilingual groups: “*If English is not spoken by group members, then interaction can be challenging.”* If communication did not go well, many feared misunderstandings and even conflicts.

Many students considered knowledge of different languages as important, but English was mentioned most as an important language in teaching multilingual groups. They evaluated their English language skills and how well they could communicate with it. Most believed that they could succeed with English, but some considered their own skills as weak, as the following student writes: “*My own language skills puzzle me, the Finnish language flows well, but others have too many sharp corners.”* In addition to this, some students wondered how to proceed if the student does not speak English either.

Many students considered a possible language barrier in multilingual groups. This could lead to the situation that pupils do not understand the instructions and the pupils do not understand each other. For example, if a pupil speaks neither Finnish nor English, the students reflected that the situation could pose a potential challenge.

The students highlighted that the uncertainty related to communication was due, at least in part, to a lack of knowledge, which led many to expect information about different cultures and religions and their customs from the upcoming course. One concern was related to the emergence of genuine interaction in the absence of a common spoken language. The language barrier and the difficulties of communicating together in terms of language or understanding of cultures and religions were linked to the fact that many pondered that they would not be able to teach the whole group with sufficient quality. For example, explaining exercises and ways of working in different languages takes a lot of time and this was considered to be out of the rest of the teacher's activities as well as taking other students into account.

One student reflected on the impact of language on his own life due to confusing situations caused by his bilinguality. He had occasionally experienced bullying when he had not spoken in the same way as other locals. He reflected that his bilingualism has increased his understanding of pupils with an immigrant background because he had experienced language-related bullying. He disclosed that bullying had had an impact on him:

*There have been places where you have been bullied because you did not know another language or grammar completely… it felt awful to come from the same country and even then, such a little thing causes such a bad experience*.

This reflection illuminates how future teachers may be able to connect their experiences on language learning and proficiency to their future professional roles and contexts.

There was also concern that teaching culturally diverse groups would bring a lot of additional work. For example, explaining exercises and ways of working in multilingual and cultural groups was thought to take a lot of time and this was considered to be out of the rest of the teacher's activities as well as taking other students into account. Many students also thought about the amount of work, if someone, for example, could not participate in a PE class with the same content due to religion. For the reasons mentioned earlier, the quality of teaching was expected to deteriorate and the amount of physical activity to decrease, which was considered as a challenge. Additional work was also considered from the perspective of the teacher's own well-being. One student was concerned about “*my own coping, and whether the quality of teaching decreases if more time is spent on giving instructions, etc.”*

Some students' thoughts on culturally or linguistically diverse groups were quite neutral. They seemed not to have particular expectations for the teaching of culturally diverse groups, or, on the other hand, particularly negative or positive thoughts. Interestingly, a few students wrote that they have no concerns at all about teaching culturally diverse groups. In their view, each group is unique, and culturally or lingually diverse groups do not differ significantly from others as the following reflection illuminates:

*I am prepared to teach those from different cultural and linguistic backgrounds and I do not think they differ in any way from other pupils. In exercise, I really often use movement as an example, so I think I can take pupils in different languages into account as well*.

As in the previous experience, the importance of visualization and demonstration emerged also in the thoughts of many other students. The experiential and embodied nature of PE was seen as a strength, so that a common spoken language is not always needed. One example of this kind of reflection comes up in the following quote: “*The lack of a common language in PE is not as challenging as in other subjects due to its functional nature.”*

Some students went even further in their thoughts concerning language; they described movement and/or sport as its own language that connects people from different backgrounds. Their reflections were related to their own experiences of how encounters in sport, for example in football, have helped to dispel prejudices toward young people from other cultures. They believed that PE in school could also play an important role in helping pupils with an immigrant background to adapt to the community and that sport could even support wider integration.

#### Synthesis and Some Critical Viewpoints

In all, students seem to be aware about how their own background has influenced their preconceptions and views. It appears that most students are, indeed, aware about the significance of equity, equality and diversity and consider these values as part of contemporary society. Many students reflected how, in their view, PE has the potential to connect people from different backgrounds and some even consider movement as a language of its own. This view may seem self-evident and widely applied in practice. It can also be explained with literature on non-verbal communication and body language, and with more recent views on embodied cognition and embodied dialogue (Anttila, 2015).

In some students, open-mindedness was connected to some level of skepticism. The position of women in religion is one issue that has given rise to critical reflections. Other students reflected that their lack of knowledge about different cultures, religions, as well as their language skills, makes them feel uncertain and also, worried about additional work that teaching culturally diverse groups may cause for them. Initially, some students expected that these courses would mend the situation by providing tools and tips that they can apply directly to their work. This phenomenon is also observed by Dervin (2015), who reports that: “when asked what they expected from a course on multicultural/intercultural education, most of my students replied that they wanted to learn about different ‘cultures' to be able to deal with ‘diverse' students” (p. 72). Dervin's study is also conducted in Finland, in the context of teacher education.

Diversity and cultural diversity can be seen as problematic concepts that may lead to categorizing individuals based, for example, on their ethnic background. For example, postcolonial scholar Bhabha (1994) speaks for the notion of cultural difference and the importance of not seeing culture as pre-given. Instead, culture must be uttered, performed, or enunciated, and through this enunciation it becomes possible to recognize cultural differences. Understanding culture as pre-given may result in “ethnic lumping,” that is, seeing individuals mainly as representatives of an ethnic group, instead of a unique person who identifies themselves with other signifiers, be it gender, political view, professional interest, or any other observable characteristic (see Rowe et al., 2018). Thus, “tools and tips” about how to encounter individuals with certain ethnic background may lead to increasing stereotypes, and thus, is a problematic approach.

Approaching each pupil as a unique individual, however, may also neglect the presence of structural inequalities that are not always apparent. Moreover, a teacher's lack of awareness of their privileges may make them blind toward differences that are not based on ethnicity or any other observable quality, but are even deeper in the societal power structures. Approaching privilege from the viewpoint of societal structures, as a question of power and human rights is important in understanding how such privilege may be at play in intercultural education (Barker, 2019). The desire to see all pupils as equal, and not consider immigrant pupils as targets of positive discrimination or any other procedure where their disadvantaged position would be seen as a predescribed fact then, is a complex issue.

While it is evident that many Finnish-born PE teacher students can be seen as privileged in comparison to many immigrants, many students have had the possibility to choose where to travel, for example. On the other hand, they also can have experiences of exclusion, inequality, and vulnerability. Their cultural encounters and their desire to reach out to and work in culturally diverse contexts may be seen as a personal enrichment and as a part of students' identity development. Some degree of “othering” (Bhabha, 1994, p.211–216) may be detected in their verbal expressions. Such accounts may reflect ignorance or naivete, and use of concepts like multiculturality or diversity vaguely. This is exactly why courses on interculturality are needed, so that students can discuss these issues and learn to formulate their thoughts in a conscious way. Moreover, these kinds of reflections can also be seen as phases in the process of transformative learning (Mezirow, 2009). We will return to this discussion in the concluding section of this article.

### Discussions, Practical Activities, and Conceptual Contents: How Emotions, Embodiment and Theory Intertwine in Students' Learning Experiences

In this section our focus is on the second research question. Our descriptive analysis is based on the second survey and the interviews. We connect students' accounts with some contents and pedagogical approaches of the courses, and shift from preconceptions to experiences, with the aim to understand what kinds of activities and contents might best support the development of future PE teachers' intercultural competence. In general, it appears that the topics of the courses, diversity and equality, seemed to have interested the students greatly, and they were considered significant and current issues. It appears that during the courses many students started to recognize their widening professional tasks in promoting equality.

According to many students, discussions were among the most important learning methods. Discussions prompted reflective dialogue between students that supported questioning, deconstructing, and rebuilding thinking. They were also central for sharing of ideas and wider learning processes related to equality. Discussions took place both under the guidance of a teacher, but also a lot during other times, for example during lunch breaks. There were some tensions between some students on how to promote equality, and for that reason equality discussions were at times very emotional for some students, as they themselves pointed out. Emotions surfaced strongly, especially when the opinions of other students were very different from their own thinking. The following quote describes the importance of emotions in discussions:

*And there were hot feelings because there was a pretty disagreement about it [equality] and it was a mentally difficult experience for me when you realized you didn't all think that way when I did. And maybe your own inner circle of friends has been terribly influenced to think so*.

Emotional discussions were also related to understanding the socio-economic background as part of equality. Some students found that not all classmates always notice how it affects their own thinking. The following reflection interestingly describes this topic:

*If you come from a prosperous family and things have always been taken care of for you… and then you come to University and quite a few get here and again you are in a good position. If you are not careful, then you can go blind to the point that not everyone has things as well*.

Before the courses, as described above, several students expressed concern about their narrow knowledge of various cultures and religions. For some students, the lack of knowledge was more specifically connected to the Islamic religion and their uncertainties about how to encounter Muslim pupils at school. In addition, the preliminary survey revealed some skepticism toward Islam as religion. Thus, a lecture on Islamic religion related to PE was added to the course. In addition to teaching the basic principles of the Islamic religion, the teacher, herself a Muslim and trained PE teacher, spoke about PE from the perspective of Muslim pupils, for example, how fasting students during Ramadan should be taken into account in PE, how Muslim pupils from different families dress, and how could the PE teacher organize changing clothes before and after the classes. One student reported having received “*important information about, for example, considering Muslims in the school world and what things belong to their religion.”*

The second course included embodied language learning. It was related to the idea that all teachers at the school are language teachers. Finland has become more heterogeneous e.g., through increased immigration. Embodied language learning (Siljamäki and Anttila, 2020) was a whole new experience for most students. In January 2021, before group teaching, the students' task was to get acquainted with the written and video materials of embodied language learning independently (Zodiak, 2021). The idea of flipped learning was used here. It refers to an approach where students acquire the necessary information before a joint meeting with the teacher's involvement. With the help of embodied language learning videos and materials, students prepared practices in small groups on the given topics, such as practicing verbs, directions, or numbers in the language chosen by the students. Students experimented teaching e.g., Spanish, Swedish, German, Swahili, or sign language to each other using movements, games and dance.

Although embodied language learning lasted for a short period of time, it seemed to have been enough to arouse interest and enthusiasm for a new kind of pedagogical approach. Some were a little confused that one could learn different languages through movement, but at the same time most students expressed that embodied language learning was an interesting and fun way to learn both language and movement simultaneously: “*Well, it was a lot of fun. A little different from what I was used to, but it was a fun experience and so… nice to note, you can really integrate language learning with movement.”* The following quote describes the thoughts of many students about embodied language learning:

*It was a really fun experience for me. I remember we laughed a lot. In my experience it was an experience that really fostered our group spirit. We also got to practice an exciting [situation], regarding speaking another language. We got this exciting practical experience of speaking another language where we all were in the same situation. It was just some language that we didn't really know. It was a nice experience and it was a really nice task*.

Interaction, group spirit and being included regardless of language became important issues as part of embodied language learning. According to the students' accounts, these were closely related to the equal encounter of pupils of diverse backgrounds. The importance of interaction and building a team spirit in supporting language learning was highlighted in the reflections of several students, of which here are two examples:

*PE has great potential in supporting language development through physical activities, body language and gestures. The physical education teacher plays an important role in the development of the interaction between pupils and between pupils and the teacher*.

*PE is a very good tool for learning a language. Awareness of the role of language in PE also helps me to address this issue in practice. Small things can have a big impact on someone's learning, motivation, and getting involved*.

In previous years, we have visited culturally diverse groups during these courses, and students have taught Finnish language to persons of different ages with an immigrant background. We have also examined students' experiences of these encounters, and we have come to the conclusion that even brief encounters with culturally diverse groups have been important and have brought about new kinds of reflections related to interculturality and equality (Anttila et al., 2018; Siljamäki and Anttila, 2020). Due to the Covid-19 epidemic, visits were not possible in 2020–2021. We asked students how they thought it affected their learning that the encounters were left out, and that courses were held entirely at the University among PE students. First, all students understood the realities of why the courses were conducted entirely at the University: “*Despite Covid-19, I felt I was learning and gaining a lot of new thinking in the course.”*

However, the students reflected on their learning experiences in the shadow of the epidemic. Students who already had previous experience teaching culturally diverse groups did not experience the lack of visits as problematic in terms of learning, than those who had no experience with groups mentioned before:

*I myself have several years of work experience as a substitute teacher, so I have also got to know culturally diverse and multilingual classes. The corona itself did not impair my learning*.

*I have worked with culturally diverse and multilinguistic groups which is why the impact is probably not great*.

On the other hand, organizing courses entirely at the University weakened learning in the opinion of many students. The sessions of embodied language learning organized between the students did not correspond to the real situation. In culturally diverse groups we would have taught the Finnish language to people who know it very little or not at all. When students taught each other different languages, there was no such challenge, as one student ponders:

*Yes, it reduced learning opportunities, because in the real situation, you can't use your own mother tongue, for example, but in a situation you have to really manage, for example, English or using body language*.

Some felt that meeting culturally diverse groups would have been important because they could have applied what they learned in lecture in practice. Many students used the term “practical experience” when considering encounters with culturally diverse groups. For teaching outside the University, the term “real situation” was also used. Practical experience was considered central to learning because it would have taught the most, it would have helped internalize the theories, concepts, and other things covered in the course, and perspectives would have expanded, as in the following reflection shows:

*It is a pity that we did not have any practical experience. The educational event would certainly have opened up a lot of new perspectives on teaching PE to culturally diverse groups*.

On the other hand, individual students felt that the practices among their own group were low-threshold activities and they had in this way also good learning experiences. Some students specifically raised their own learning needs when considering the lack of practical experience. They thought that meeting culturally diverse groups would have lowered their anxiety or it would have brought additional motivation. The emphasis of these students is that they would have liked to learn from persons from different backgrounds. Here is one example:

*It would certainly have been really rewarding to get to meet culturally diverse and multilingual classes. I think there would have been a lot of interesting and instructive conversations with them*.

However, most of the students were positive despite the lack of practical experience and they reflected that they need practical work experience in any case in order for the renewed ideas and perceptions to be transferred to pedagogy and teacher work in the future.

#### Synthesis and Some Critical Viewpoints

As the courses progressed, it appears that students' awareness of the significance of equity, equality, and diversity increased. The students also reflected about PE teachers' widening professional roles and tasks. These reflections can be seen as a sign of expanding professionalism (Siljamäki and Anttila, 2020; Sutela et al., 2021; Westerlund and Gaunt, 2021).

Students seemed to value discussions in and outside the classes greatly. Discussions, of course, are an important form of reflection and especially valued in the context of higher education. Interestingly, emotional discussions were also mentioned. Here, it is possible to see a connection to transformative learning that, according to Mezirow (2009) begins with a disorienting dilemma, and that Mälkki (2010) explains as having to do with so-called edge-emotions. Even before the courses, some students reflected on their feelings of uncertainty, which can already be considered as a disorienting dilemma that can potentially lead to a process of transformative learning. As an example of a habit of mind Mezirow mentions ethnocentrism that refers to “the predisposition to regard others outside one's own group as inferior, untrustworthy or otherwise less acceptable” (Mezirow, 2009, p.93). Having a positive experience with such groups may lead to change in point of view, but fail to change the habit of mind. Thus, it is difficult to evaluate whether or not students' habits of minds have changed, and even more difficult to foresee how this change may influence their pedagogical practice.

As Mezirow suggests, positive experiences may be a significant element in changing habits of mind. According to Mälkki (2010), however, the role of emotions, more specifically, edge-emotions, are crucial in transformative learning. Moreover, meaning perspective also involves a social dimension. It is formed through socialization and affected by culture and significant others. This social construction of meaning perspective is connected to intersubjectivity of personal meanings, which then enables interaction, acceptance, and integration to groups and cultures. The bond between people is based on shared meanings tends to be maintained with the help of emotions, and threats to social dimension may arouse the uncomfortable edge-emotions (Mälkki, 2010, p.32).

In our previous study, we found that PE teacher students had mixed feelings toward visiting an asylum seekers' center. After the visit, the emotions were, however, very positive. The single visit that included embodied, or kinesthetic, language learning workshops seemed to have been a transformative learning experience for many where a disorienting dilemma and edge-emotions created a sudden shift in students' meaning perspectives. We concluded that field experiences are a significant element in transformative learning, and in developing students' intercultural competence (Anttila et al., 2018). As we mentioned earlier, the pandemic made encounters with culturally diverse groups impossible. Based on students' accounts in this current study, it seems that students valued practical experience even when it took place at the University, with their peers. It is also quite evident that they regretted not having the possibility for actual field experience. However, disorienting dilemmas and edge-emotions have been present in these courses even if in a less distinct way. Thus, it is possible to infer that transformative learning, albeit in a cumulative—vs. sudden—sense has taken place during these courses.

Students' experiences regarding practical activities especially on embodied language learning support the view on embodied learning as both supportive of transformative learning, as well as something that enhances social cohesion among the students. Moreover, practical activities seem to support internalizing academic content of the courses.

On a critical note, based on our research it is impossible to foresee whether or not the development of intercultural competence results in changes in student teachers' pedagogical practice, and how permanent the changes in their habits of mind and subsequent actions are. Also, our interpretation regarding the nature of students' learning processes as embodied and transformative might be biased as we, as our interest in embodiment, embodied knowledge, cognition, and learning has framed our research for several years. We are inspired by the embodied nature of emotions, the role of emotions and embodiment in reflective practices, and in developing higher education toward more holistic pedagogical practices (Mälkki, 2010; Sööt, 2018; Sutela et al., 2021).

### Expanding Views: How Students Demonstrate Their Privilege, Positionality, and Responsibility as Future PE Teachers

Focusing now on the third research question, we are detecting signs of change in students' conceptions. After both courses, students were asked to reflect and describe what they had learned during the courses, and how their conceptions may have changed. In general, many students described that their own values and perceptions became clearer and their awareness about the complexity of interculturality had increased. Many students said that their preconceptions did not change so much, but they expanded. By the end of the courses, they seemed to be more convinced than in the beginning of the courses about the importance of the themes of equality and diversity in physical education teacher training. One of the most important things learned in the course seemed to be an aspiration to provide equal opportunities for all students at school as a future PE teacher. An example of this is given in the following quote: “*The fact that I, as a teacher, play an important role so that every student feels equal.”* This is another sign of expanding professionalism discussed above. These kinds of statements also refer to the idea that every teacher is a language teacher, and that movement and bodily expression is a language. In the interview, one student reflected the change his preconceptions in this way:

*I don't know if perceptions changed, but yes, it just kind of made me think from the teacher's point of view that you have to be able to take those things into account… I didn't think about these things in the past… or I can't even think about all that has to be taken into account if there are culturally diverse classes*.

Also those students who had earlier experiences related to culturally diverse groups described that their conceptions had broadened. They reflected that they found new perspectives on equality. Some of these students pointed out that they had perceived diversity and equality more narrowly or had not come to think of some relevant things before the courses. The students reflected on the expansion of their own thinking or changed attitudes for example in the following ways:

*My world of thought has expanded. I have learned to identify situations where, for example, I have inadvertently maintained stereotypes*.

*I now have a broader perspective on cultural diversity and I can address things by their real names*.

At the beginning of the course, as noted earlier, many students hoped to learn about different cultures, religions and languages in order to be able to work with diverse groups. There were few such comments at the end of the course. The courses did not include teaching of the “different cultures or religions and their customs,” with the exception of the lecture concerning the teaching of Muslim pupils. One student reflected on the lecture in this way: “*At least I have learned from Muslim culture that there can be very big differences in the way how different families think and act.”*

This reflection highlights how interculturality began to appear more complex after the courses than many students had previously thought. This seemed to be one of the most important change in conceptions for many students, as the following quotes from the closing survey bring out:

*A lot of my own thoughts have revolved around such things that there is no one right answer to anything. It feels like that idea becomes stronger the wiser I become! I also have an increased understanding of others' choices and opinions*.

*I have learned that there is always more to learn about interculturality; all the time there is more knowledge and opportunities to consider different things from new perspectives in terms of different forms of cultures both in other people and in oneself. The courses have also made it very clear how individual an experience as social a matter as culture can be. Interculturality can also require the ability to talk about difficult things or feelings*.

There were a lot of similar reflections after the courses, which considered an increase in criticality in one's own thinking and a new kind of awareness of issues related to the encounter of diverse groups. Some of the students also reflected on the things they learned during the courses more broadly, in terms of their own lives. For example, they began to use concepts covered in the course to learn to articulate their past experiences related to racism that they have encountered. This may be a significant learning experience for them as future teachers. One student pondered that she had previously had difficulties in identifying racism. After the courses she wondered if it would be easier for her to recognize racism in future and how she would act in such a situation:

*So maybe I'll be more specific in the future, at least if someone comes to tell me, then I have to somehow take it seriously, at least those feelings…. the fact that another person has aroused such feelings about it. So it is still important to be heard and talked about*.

These kinds of reflections have also helped them to understand immigrants coming to Finland, as in the words of one student:

*It may have made it easier to understand that even though immigrants come to Finland, it is the country of origin where you grew up, it has shaped them and we are here in Finland and we have this country's norms that affects us. So I understand the differences better and also the factors that explain them*.

One student, who has lived in two different countries, pondered the significance of culture in many ways during the courses, for example, the importance of culture in relation to embodiment and body language: *Well at least that's how much that culture affects a person. How much does it mean, for example, how do you interpret someone's smile or how do you interpret someone's body position. Somehow it has given me quite a bit of understanding about it*.

When asked how equality and interculturality have been taken into account in education, several students said that they have only been addressed in the courses covered by this study. Some students mentioned that in the context of dance classes and in swimming there have been discussions on how culturally and religiously diverse groups are taken into account. Many students pointed out that equality should not only be a matter to be taught and addressed in separate courses, but should be part of the whole teacher education. In their view, equality should appear as a red thread in different sports environments as well as in the other courses of teacher training of physical education.

*I personally consider equality issues to be extremely important and it would be a great opportunity at the University to influence the understanding of our students. I would like equality to be extended to all courses*.

Some went even further than the previous idea: in the opinion of them equality should be not only in the teacher training but also as a compulsory general education course for all University students, as one student pointed out: *In my opinion, a course like this could very well be among the compulsory studies in all fields, as it would increase equality and reduce prejudice*.

In addition to the possibilities of embodied language learning, students also considered other ways to integrate language teaching into physical education. Students described their insights on the various ways in which they can be a PE teacher and at the same time language teachers. In the following quote one student highlighted this in the following way:

*My understanding of the PE teacher as a language teacher was strengthened. Each teacher acts as a language teacher for their own subject. I also realized how good opportunities there are for learning a language through exercise and movement. Functionality combined with language learning reinforces memorization*.

Some students saw equality as a broader phenomenon; equality means, for example, that each student is offered movements or practices appropriate to his or her skill level. If equality is understood in this way, it is present in many courses, according to students. Students' accounts also include expectations for university teachers related to equality. The students hoped that all faculty members would be involved in the dialogue. They emphasized that change requires all parties: teachers must also be prepared to critically reflect on their own perceptions, values and ways of acting in order to take equality issues into account in education. Students also felt that the faculty should review some traditions and norms as well as ways of speaking that were previously considered accepted. For example, comments that differentiate people on the basis of their origin, were considered particularly harmful. According to the students all teachers need to be constantly vigilant and update their knowledge related to interculturality and more broadly to equality. In the words of one student:

*Yeah, keep that kind of open-mindedness in not climbing that kind of ivory tower as a professor either… then there is an opportunity to make an impact, then (s)he would use own lot correctly*.

Some students raised the issue of generations in attitudes related to equality, as in this account:

*Perhaps it is precisely the fact that, generations, many things are slowly changing. So then I feel like during my generation a change has happened in a really short time so that interculturality and equality in general have become very important things*.

#### Synthesis and Some Critical Viewpoints

It is evident that students' views on equity, equality, and interculturality expanded during the courses. They reported having become more aware of the complexity of these issues. Their ability to reflect their own views critically seems to have increased. These developments are quite expected and in line with the learning outcomes designed for these courses. The purpose of our study, however, is not merely to evaluate how well the courses succeeded in achieving the learning outcomes, but to dig deeper into students' learning experiences and the nature of change that has occurred.

As we mentioned above, discussions and practical activities seem to have been most meaningful for the students. Intertwinement of emotions and embodied activities with conceptual and theoretical content seem to work toward change toward expanding views and critical reflection. Thus, changes in habits of mind (Mezirow, 2009), seem to require not only acquisition of new knowledge and detailed information, but also emotional and embodied engagement. The ability to encounter and deal with edge-emotions (Mälkki, 2010) and to participate fully in embodied, collaborative activities where learning engages the whole person (Anttila, 2015) may have a key role in supporting the process of transformative learning. The possibility for field experience that was now lacking due to Covid-19, seems crucial in this regard. Working practically among peers and engaging in discussions, however, seemed to give opportunities for edge-emotions to appear, at least to some extent. Our interpretation on the significance of emotions and embodiment may, of course, be questioned and the expanding of students' views may have their origin outside these courses and the University. Several students refer to a wider context than PE. They reflect that the topics of these courses should be included in all teachers' education, or even compulsory for all University students, and underlying themes in all University education. Moreover, some students demand that all University faculty should be able to address these issues with more awareness on how they speak and teach. It is possible to interpret that a generation gap between University students and faculty exists, and that students, in general, are more aware about these issues than their teachers.

Opportunity to learn about and from other cultures could, in general, be even interpreted as cherry picking experiences that benefit one's professional and personal development. From this viewpoint, different cultures can be seen as a source of wealth, as long as the experiences stay within the limits of one's comfort zone. While student accounts in our study do not give rise to this kind of interpretation, our analysis does not confirm that deep and permanent changes in students' meaning perspectives have taken place. A new follow-up study would be needed for making further interpretations on the nature of change in the students' conceptions, and in seeing how such change might inform their pedagogical practices.

We wonder how it might be possible to see migrants—as well as anyone representing a minority or a marginalized group–as equal individuals with unique needs, capabilities, and desires. The question, thus, remains: whose point of view is at stake when student teachers' intercultural competence is being developed? Is it possible to provide student teachers with both meaningful, personal learning experiences and a heightened sense of solidarity that includes critical self-reflection toward their privileged positions?

## Conclusion

In this article we have sought to highlight the preconceptions of the PE teacher students concerning interculturality, language awareness and equality as well as the students' descriptions about how these conceptions may have changed in the context of the equality and equity courses. In addition, we have shed light on what kind of learning experiences the students had in connection with the courses. Among the most significant changes in preconceptions is related to understanding how important it is to interact with pupils from various backgrounds on a case-by-case basis (e.g., Friedman and Berthoin Antal, 2005; Nastasi, 2017). In our previous articles we have already emphasized the importance of situational awareness in developing PE teacher students' intercultural competence (Anttila et al., 2018; Siljamäki and Anttila, 2020). Friedman and Berthoin Antal (2005) use the term negotiation reality and emphasize that negotiation reality generates the necessary cultural knowledge for situations as they arise, and from this knowledge constructs fruitful action strategies. This strategy is beneficial because a person rarely has a huge amount of knowledge about different cultures or religions. On the other hand, this strategy is demanding, because individuals should have an active awareness of how their own background influences their perceptions and behavior or e.g., an ability to find out different ways of seeing and doing things with others or solving the problem situations.

Before the courses several students expressed that they expected to be taught knowledge about different cultures and religions. Afterwards, only a couple of students mentioned this need to gain specific information related to cultures and religions so that they would know how to act in an appropriate manner with all pupils. The courses did not include such information, except for one lecture related to PE for Muslim pupils. Despite this, many students reported that they had learned a lot about different cultures and religions. How to interpret this apparent discrepancy?

One way to understand this is that the students acquired conceptual tools with which they can address these issues in their future work as physical education teachers. Through the intertwinement of concepts, knowledge, and practice, the students may have more confidence to speak more accurately and precisely about issues and phenomena related to cultures, religions and interculturality than earlier. One of the most important insights for students was to never assume anything in advance. Thus, it is crucial to always ask pupils themselves or their parents about how they embody the norms and values of culture or religion that they share and how they could be taken into account in PE. This is, in our view, what situational awareness means in practice. We suggest that this kind of increase in situational awareness has a key role in reinforcing students' experience of learning bout different cultures.

As we have already discussed, the connection between conceptual knowledge and practical experience seems to be important in the process of developing future teachers' intercultural competence. We see that embodied learning, in this context, has a deeper meaning than being a pedagogical approach for teaching PE in culturally diverse classes. Embodied learning connects the conceptual, reflective, emotional, and affective levels. It is associated with the increase of bodily awareness and this ability may support student teachers in recognizing and dealing with so-called edge-emotions in both discussions and in practical situations. This kind of holistic approach, in our view, may be a key in developing teachers' intercultural understanding and competence.

This aim may require taking culture out of interculturality, a move that for example Dervin strives for. He (2015) writes that “culture may not always be the main (explicit) emphasis in intercultural interaction” (p. 74). The question of power, indeed, becomes central in this post-intercultural education, as Dervin asks: “Who decides who is allowed to identify in complex or limited ways?” (Dervin, 2015, p.74). He also emphasizes the significance of being aware of complex, intersecting positions that may be collective, individual, and sometimes contradictory. This is partly why teaching the features of different cultures as a part of teacher education is problematic. It may support and even increase stereotypes toward other cultures, simplify them and at worst, put some cultures in a lower position (Kumaravadivelu, 2003, 2008; Dervin, 2015; see also Miettinen, 2020). Thus, intercultural education should not be about “how to deal with diverse students.” It may also be important to think beyond culture in intercultural education, and to focus on each person as a unique human being.

The research area of intercultural competence is wide-ranging, and we consider it important to continue to work with this topic. A wider recognition of structural inequalities and racism is needed. Understanding that societal norms and power structures influence teachers even when they personally declare being antiracist, is crucial. Research is also needed as a basis for a critical review of curricula in higher education.

## Data Availability Statement

The raw data supporting the conclusions of this article will be made available by the authors, without undue reservation.

## Ethics Statement

The studies involving human participants were reviewed and approved by the University of the Arts Helsinki's Ethical Review Committee. The participants provided their written informed consent to participate in this study.

## Author Contributions

MS and EA have designed the study together. MS collected the material and analyzed it with EA. EA took the responsibility for writing the methods. MS wrote the first version of the result section. EA was first responsible for interpretations, and later MS has approved them. During the working process, both authors have worked together with the manuscript and approved the submitted version.

## Funding

This research has been undertaken as part of the ELLA-project (Grant no. 202007315) (Embodied Language Learning through the Arts), funded by the Kone Foundation (01/2021–12/2024).

## Conflict of Interest

The authors declare that the research was conducted in the absence of any commercial or financial relationships that could be construed as a potential conflict of interest.

## Publisher's Note

All claims expressed in this article are solely those of the authors and do not necessarily represent those of their affiliated organizations, or those of the publisher, the editors and the reviewers. Any product that may be evaluated in this article, or claim that may be made by its manufacturer, is not guaranteed or endorsed by the publisher.

## Footnotes

^1^The statistics are based on Statistics Finland's (2020) figures. According to them, at least one of the parents of a person with an immigrant or foreign background was born outside Finland. The person may have been born either abroad or in Finland. When students in the study use the term immigrant, we are certainly not able to say how they themselves perceive the concept.

^2^The concept of culturally diverse groups/classes in this context refers to persons with an immigrant background and minorities living in Finland, such as the Swedish-speaking Finns, Sami, Romani, as well as the persons from different religious communities.

^3^The term “student” refers here to a PE student and the word “pupil” is used as a term of reference for younger learners at school, e.g., in basic education.
